# Assessment of chemotherapy-induced peripheral neuropathy using the LDI_FLARE_ technique: a novel technique to detect neural small fiber dysfunction

**DOI:** 10.1002/brb3.354

**Published:** 2015-05-26

**Authors:** Sanjeev Sharma, Ramachandran Venkitaraman, Prashanth R J Vas, Gerry Rayman

**Affiliations:** 1 Diabetes Research Unit, The Ipswich Hospital NHS TrustIpswich, UK; 2 Department of Clinical Oncology, The Ipswich Hospital NHS TrustIpswich, UK

**Keywords:** CIPN, EORTC, LDI_FLARE_, nerve conduction velocity and amplitude, QLQ-CIPN20

## Abstract

**Introduction:**

The diagnosis and quantification of chemotherapy-induced peripheral neuropathy (CIPN) remains a challenge. Conventional methods including quantitative sensory testing (QST), nerve conduction tests, and biopsy are unable to detect subclinical changes, and do not consistently correlate with severity of patients' symptoms and functional impairment. This study aims to determine the utility of the LDI (laser Doppler imager) _FLARE_ technique in the diagnosis of CIPN and whether it correlates with symptom severity.

**Materials and Methods:**

We assessed 24 patients with established CIPN [12 due to platinum analogs (P_A_) and 12 to Taxanes (T_X_)] and 24 matched healthy controls (H_C_). All underwent neurophysiological examination including vibration perception threshold (VPT), sural nerve amplitude (SNAP) and conduction velocity (SNCV), LDI_FLARE_, and fasting biochemistry. The QLQ-CIPN20 questionnaire was used to assess symptom severity.

**Results:**

H_C_, combined chemotherapy (C_G_), P_A__,_ and T_X_ groups were matched for age, sex, BMI, and blood pressure. The LDI_FLARE_ was significantly reduced in C_G_ compared to H_C_ (*P* =< 0.0001), whereas SNAP (*P* = 0.058) and SNCV (*P* = 0.054) were not. The LDI_FLARE_ correlated with the QLQ-CIPN20 symptom scores in all three categories namely, C_G_ (*P* =< 0.0001), P_A_ (*P* = 0.001) and T_X_ (*P* = 0.027) whilst, VPT, SNAP, and SNCV did not.

**Conclusion:**

Our findings suggest that the LDI_FLARE_ technique is more helpful in confirming the diagnosis of CIPN in patients with distal sensory symptoms than current commonly used methods. Moreover, this novel test fulfils the unmet need for a diagnostic test that relates to the severity of symptoms. This may be useful in quantifying early changes in small fibre function indicating early CIPN.

## Introduction

Chemotherapy-induced peripheral neuropathy (CIPN) is the most common neurological complication of cancer treatment (Kannarkat et al. 2007) often causing severe debilitating symptoms leading to dose reductions or premature terminations of chemotherapy. Moreover, the impact on quality of life can be profound with many patients left with permanent neuropathic pain.

The symptoms of CIPN are varied and related to the specific toxicity of the class of agent involved. Presentation varies from exclusive sensory impairment to mixed sensorimotor neuropathy and less commonly, pure motor neuropathy. Distal axonopathy is the commonest clinical presentation and its features include progressive, distal symmetrical length-dependent sensory neuropathy manifesting as paraesthesia, allodynia, and hyperalgesia in a classic stocking followed by glove distribution (Hausheer et al. 2006). As there is no specific, reliable diagnostic test of CIPN, the diagnosis is currently based on clinical criteria: a combination of clinical symptoms and signs, electrophysiological measurements (EPM) and quantitative sensory tests (QST). However, conventional EPM's such as nerve conduction velocity (NCV) and electromyography (EMG) may be normal in the presence of small nerve fibre damage (Sissung et al. 2008) and are therefore considered complimentary rather than diagnostic (Kaley and Deangelis 2009). Moreover, they show poor correlation with severity of clinical symptoms (Berger et al. 1997) and have failed to demonstrate any clear benefit over simple clinical symptom-scoring systems for monitoring the condition (du Bois et al. 1999; Mileshkin et al. 2006). Similarly, QST techniques like vibration perception threshold (VPT) are not reliable in the assessment of CIPN and do not always correlate with severity of symptoms (Forsyth et al. 1997). In addition to the above methods, there are several published clinician-rated methods for assessing CIPN that can be used either alone or in addition to EPM or QST techniques to grade the severity. Of these, the most widely recognized include the National Cancer Institute-Common Terminology Criteria (NCI-CTC version 3), WHO, Eastern Cooperative Oncology Group (ECOG) and Ajani criteria (Miller et al. 1981; Ajani et al. 1990; Trotti et al. 2003; Hausheer et al. 2006). These are based on a combination of patients' subjective reporting of severity of symptoms and physicians' objective assessment of a combination of sensory and motor neurological signs. Whilst it is beyond the scope this submission to examine these in detail, there is extensive literature citing their relative shortcomings which include overdependence on subjective symptoms and a significant variability in grading between examiners (Postma et al. 1998; Postma and Heimans 2000; Windebank and Grisold 2008). Indeed, most CTC scores mix impairment, disability, and quality of life measures which could lead to misinterpretation of the results and under or overestimation of the effect. Lastly, these scales are based on the Likert theory and are subject to central-tendency bias – that is, scorers and responders avoid extreme response categories (Cavaletti 2009).

Given these limitations, there is clearly an unmet need for a sensitive and reliable test to diagnose and assess the progression of CIPN. The LDI flare (LDI_flare_) technique is a recent noninvasive sensitive method for assessing small nerve fibres (SNF) function that has been used to detect small fibre neuropathy in people with diabetes (Krishnan and Rayman 2004). The method measures the size of the axon-reflex-mediated neurogenic flare after heating of the dorsal foot skin. The aim of this study was to evaluate the validity of the LDI_FLARE_ technique when compared to both subjective and objective measures of CIPN severity. The former included EORTC QLQ-CIPN20 (European Organisation for Research and Treatment of Cancer Quality of Life Questionnaire-Chemotherapy-induced Peripheral Neuropathy 20 module) questionnaire whilst objective measures for neurological severity included vibration perception threshold (VPT), sural nerve conduction velocity (SNCV), and amplitude (SNAP).

## Materials and Methods

### Ethical approval

This study was conducted in accordance with the Declaration of Helsinki and approved by the ethics committee of the NRES Committee East of England – Norfolk, UK (REC reference: 13/EE/0162).All patients provided a written informed consent.

### Study design and participants

This is a proof of concept study involving patients with previously established diagnosis of CIPN, all of whom had previously completed their chemotherapy regimens at least a year prior to assessment. To the best of our knowledge, we are not aware of any published literature citing the use of the LDI_flare_ technique in the assessment of CIPN and hence knowing the reproducibility of the techniques and based on our previous work mainly in diabetic peripheral neuropathy, we estimated that an sample size of 24 patients would be adequate to demonstrate the reliability of the technique in detecting small fibre dysfunction in patients with established CIPN.

We selected the two most commonly affected groups of patients', namely those who had received platinum (oxaliplatin, carboplatin, cisplatin) analogs (P_A_) or Taxanes (docetaxel, paclitaxel), (T_X_). Based on their clinical profile and treatment regimes, 15 patients in each group were randomly selected initially from the hospital oncology CIPN database at The Ipswich Hospital NHS Trust, (Ipswich, UK) and recruited into the study after acceptance of the formal invitations. All selected patients had either P_A_ or T_X_ as their main chemotherapeutic drug and did not receive any combination of additional neurotoxic chemotherapeutic agent. All subjects had stable cancer disease at the time of assessment and had completed their treatment regime at least 12 months prior to recruitment. None of the participants were on any active cancer management except routine surveillance as per their oncological diagnosis.

For comparison, an equal number, that is, 24 of age-matched healthy controls (H_C_) were studied. They were recruited by invitation in hospital newsletters and local press and the majority comprised hospital staff members.

### Assessments

All subjects underwent detailed neurological examination of sensory and motor nervous systems as per Neuropathy Disability Score (NDS) (Boulton 2007) guidelines, including bedside temperature perception (hot and cold using tuning fork with beaker of ice/warm water), 10 g of mono-filament pressure and 40 g pain (pin-prick) sensations using Neuropen™ (Owen Mumford, Oxford, UK), achilles deep tendon reflexes, and proprioception. Tests for large fibre function included VPT and sural nerve EPM parameters. VPT was measured in both halluces using a neurothesiometer (Horwell Scientific Laboratory Supplies, Wilford, Nottingham, U.K.) following the established method of limits (Shy et al. 2003) while sural nerve amplitude (SNAP) and conduction velocity (SNCV) was estimated using the NC-stat® ¦ DPNCheck™ device (NeuroMetrix Inc, Waltham, MA) (Perkins et al. 2008). Recent studies have shown that this device demonstrates excellent reliability and comparable accuracy in relation to conventional nerve conduction studies (Perkins et al. 2006; Lee et al. 2014). Our recent work with this device in diabetes neuropathy states have also demonstrated excellent correlation with clinical neuropathy scores and the LDI_FLARE_ method in various severities of diabetes neuropathy (Sharma et al. 2014).

Fasting biochemical assessment was performed in all subjects to exclude conditions associated with neuropathy, including diabetes mellitus, hypothyroidism, and hypertriglyceridemia. Six patients in total were excluded due to biochemical features of diabetes (three), impaired glucose tolerance (one), subclinical hypothyroidism (one), and raised triglycerides (>1.7 mmol/L; one). For the grading of CIPN severity, we used the EORTC QLQ-CIPN20 (European Organisation for Research and Treatment of Cancer Quality of Life Questionnaire-Chemotherapy-induced Peripheral Neuropathy 20 module) questionnaire (Postma et al. 2005) as current evidence recognizes this as a validated ‘patient-oriented’ evaluation of CIPN symptoms and functional impairment (Wolf et al. 2012; Lavoie Smith et al. 2013). The current version of CIP20 is a 20-item patient self-reported CIPN-specific questionnaire that includes three subscales assessing sensory (nine items), motor (eight items), and autonomic symptoms (three items) with each item measured on a 1–4 Likert scale (1 being “not at all” and 4 being “very much”). The minimum score is 20 whilst the maximum score is 80 and a higher score indicates a greater severity of subjective symptoms and functional impairment.

All H_C_'s underwent the same biochemical and neurological assessments.

### The LDI_FLARE_ technique

**Figure 1:**
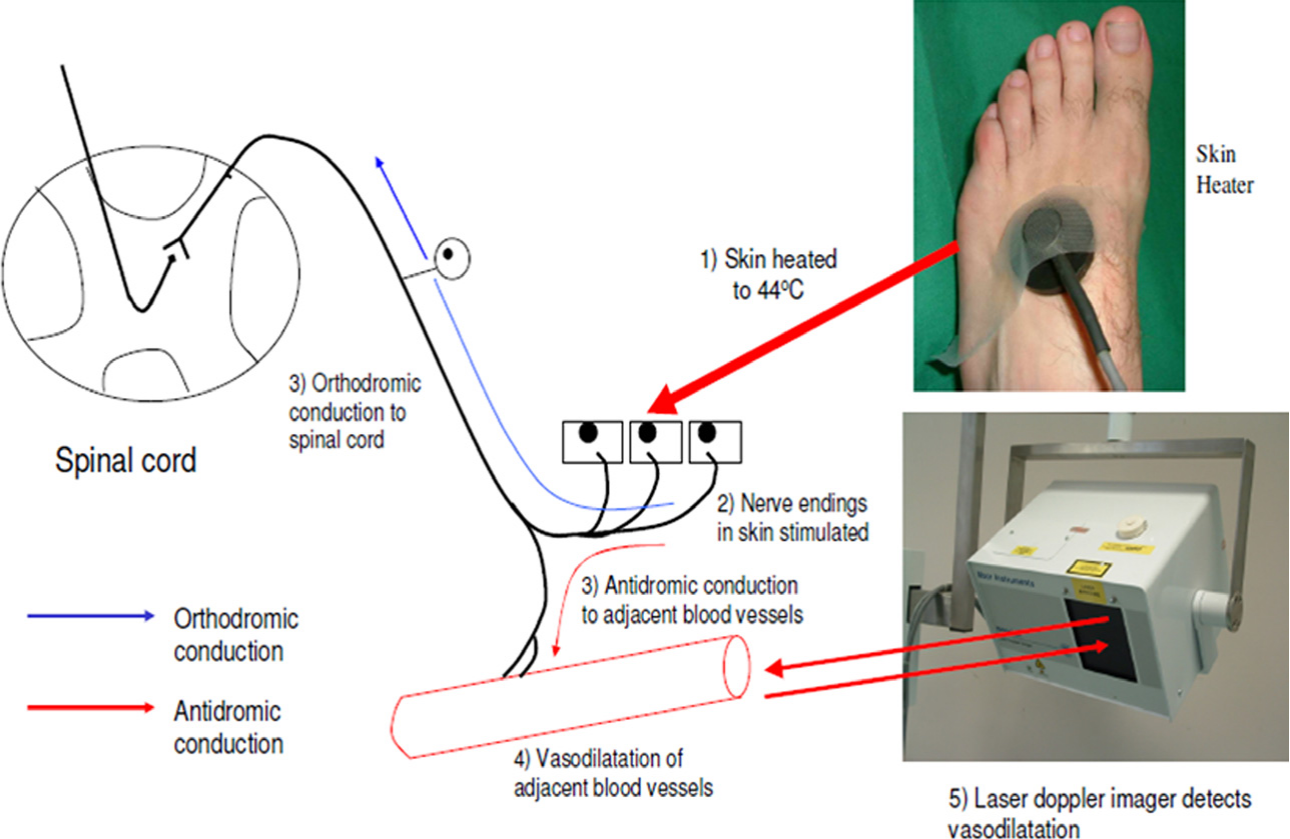
Schematic representation of the principle of the LDI_FLARE_ technique which measures the nerve-axon-related vasodilatation in response to heating of the foot fore skin.

**Figure 2:**
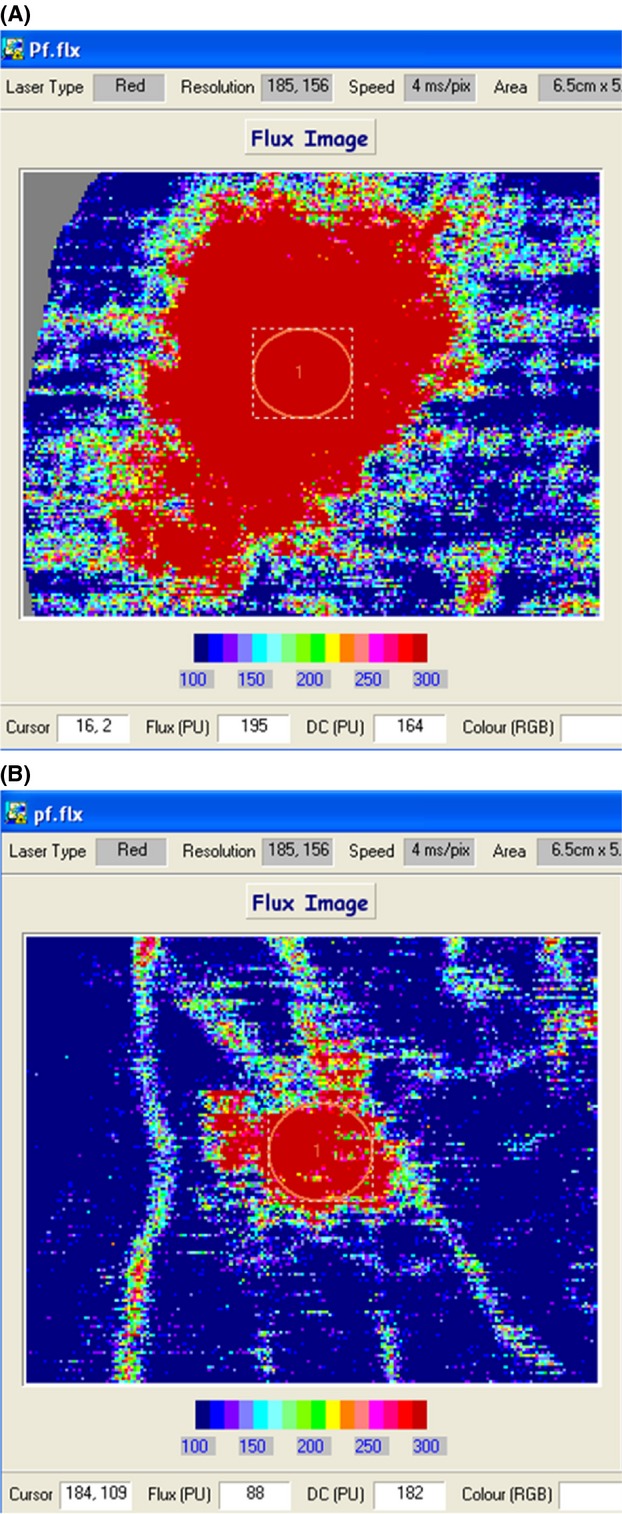
The LDI_FLARE_ in a H_C_ and a patient with CIPN. (A) A normal LDI_FLARE_ in a H_C_ (size of 8.3 cm^2^), while (B) The reduced LDI_FLARE_ in a patient with severe CIPN after carboplatin treatment (LDI_FLARE_ size of 1.7 cm^2^).

Small nerve fibre (SNF) function was assessed using the LDI_flare_ technique as previously published (Vas and Rayman 2013b). This method involves heating of the dorsal foot skin to 47°C using a 1 cm^2^ heating probe (Fig.1) and measuring the resultant nerve-axon-related hyperemic response, using a laser scanner. The size of the hyperemic response in cm^2^ is quoted as the “LDI_flare_” (Fig.2). Flare size relates to SNF function; a small flare equates to reduced SNF function. The technique has been used to detect C-fibre dysfunction as an early marker of small fibre neuropathy in type 2 diabetes (Krishnan and Rayman 2004) and impaired glucose tolerance (IGT) patients (Green et al. 2010). Moreover, using the LDI_flare_ technique, we also demonstrated that glycaemic burden and the presence of microvascular complications are associated with small fibre dysfunction in type 1 diabetes (Vas et al. 2012). In relation to the present study, we recently demonstrated that the LDI_flare_ size shows an age-dependent decrease (Vas and Rayman 2013a) necessitating careful age matching of comparison groups.

### Statistical analysis

Statistical analysis was performed using SPSS (version 22 for Windows, Armonk, NH) and StatsDirect (StatsDirect Ltd. version 3.0. StatsDirect statistical software. http://www.statsdirect.com. England: StatsDirect Ltd. 2013) 3. Clinical characteristics were expressed as mean ± SD. Difference in categorical variables were assessed in two- and three-group comparisons using the *χ*^2^ test while differences in continuous variables were assessed using ANOVA. The linear associations between QLQ-CIPN 20 scores and LDI_flare_ outcomes were assessed using the Spearman rank correlation method. To assess the independent effect of age on various small and large nerve fibre assessment methods, we used linear regression in a multivariate model along with BMI as a covariable. We explored the possibility of nonlinear relationships by way of standard transformations (including powers, roots, logarithms, and polynomial), but none offered any advantage over linear regression or Spearman correlations. The coefficient of variation (CV) for LDI_flare_ is conservatively estimated at 20% (actual CV's for LDI_flare_ is 8.7%).

## Results

The clinical characteristics of the groups studied are shown in Table 1990. Comparisons were made between H_C_ and Combined groups (C_G_), each comprising 24 matched subjects and between the individual P_A_ and T_X_ subgroups and are shown in Table 1997.

**Table 1:** Clinical characteristics, neurophysiological outcomes, and QLQ-CIPN20 scores and their significance

	Healthy controls (H_C_)	Combined groups (C_G_)	Platinum analogues (P_A_)	Taxanes (T_X_)
*N*	24	24	12	12
Age (years)	63.75 ± 8.40	65.42 ± 7.90	63.67 ± 11.39	67.17 ± 8.72
Female/Male sex	10/24	10/24	3/12	7/12
Duration since completed chemotherapy (months)	–	13.1 ± 1.0	12.5 ± 0.9	13.6 ± 1.1
BMI (kg/m^2^)	28.91 ± 5.30	29.26 ± 3.45	28.95 ± 2.85	29.57 ± 4.06
Systolic blood pressure (mm Hg)	121 ± 19	135 ± 18	136 ± 8	133 ± 24
Diastolic blood pressure (mm Hg)	69 ± 11	85 ± 7	83 ± 9	86 ± 6
LDI_flare_ (cm^2^)	6.53 ± 0.75	3.75 ± 1.68	2.72 ± 0.54	4.79 ± 1.99
Neuropathy disability score (NDS – maximum score 10)	0	5.79 ± 1.32	6.58 ± 0.99	5 ± 1.08
Large fibre neuronal parameters				
Vibration perception threshold (volts)	8.50 ± 2.50	21.39 ± 8.03	23.06 ± 5.57	19.71 ± 4.21
Sural nerve amplitude potential (SNAP – *μ*V)	15.54 ± 3.13	10.13 ± 3.12	9.92 ± 4.43	10.33 ± 3.67
Sural nerve conduction velocity (SNCV – m/sec)	52.08 ± 5.52	42.04 ± 9.11	35.83 ± 7.91	48.25 ± 5.15
QLQ-CIPN20 score	–	38.67 ± 9.27	48.08 ± 7.40	29.25 ± 7.21

*N*, number of patients; BMI, body mass index; LDI_FLARE_, laser Doppler imager flare; EORTC QLQ-CIPN20, European Organisation for Research and Treatment of Cancer Quality of Life Questionnaire-Chemotherapy-induced Peripheral Neuropathy 20 module. Significance = *P* < 0.05.

**Table 2:** Spearman rank correlations between healthy controls (H_C_) and Combined groups (C_G_), each comprising 24 matched subjects and between the individual P_A_ and T_X_ subgroups

Spearman rank correlations	*P* for C_G_ vs. H_C_	*P* for P_A_ vs. H_C_	*P* for T_X_ vs. H_C_	*P* for P_A_ vs. T_X_	Correlation with QLQ-CIPN20 score
Age (years)	0.87	0.56	0.41	0.47	–
Female/Male sex	0.50	0.45	0.55	0.10	–
Duration since completed chemotherapy (months)	–	–	–	0.98	–
BMI (kg/m^2^)	0.63	0.27	0.33	0.55	–
Systolic blood pressure (mm Hg)	0.10	0.09	0.21	0.33	–
Diastolic blood pressure (mm Hg)	0.09	0.07	0.12	0.58	–
Neuropathy disability score (NDS)	–	–	–	0.06	0.10
LDI_flare_ (cm^2^)	**<0.0001**	**<0.0001**	**0.005**	**0.007**	<0.0001*
Large fibre neuronal parameters					
Vibration perception threshold (volts)	**0.002**	**0.019**	**0.020**	0.11	0.67*
Sural nerve amplitude potential (SNAP – *μ*V)	0.058	0.43	0.19	0.29	0.33*
Sural nerve conduction velocity (SNCV – m/sec)	0.054	0.067	**0.45**	0.25	0.42*
QLQ-CIPN20 score	–	–	–	**<0.0001**	–

P_A_, Platinum analogues; T_X_, Taxanes; *N*, number of patients; BMI, body mass index; LDI_FLARE_, laser Doppler imager flare; EORTC QLQ-CIPN20, European Organisation for Research and Treatment of Cancer Quality of Life Questionnaire-Chemotherapy-induced Peripheral Neuropathy 20 module. Significance = *P* < 0.05 and where significant are shown in bold.

* As Combined group (C_G_).

H_C_ subjects did not significantly differ from C_G_ patients in terms of age, sex, BMI, and both systolic and diastolic blood pressure. As expected, VPT was significantly higher in C_G_ compared to the H_C_ group (*P* = 0.002). However, both SNAP and SNCV, though lower, were not significantly reduced in C_G_ (*P* = 0.058 and *P* = 0.054, respectively) when compared to H_C_. In contrast, SNF function assessed by the LDI_FLARE_ method was significantly reduced in the C_G_ group (6.53 ± 0.75 vs. 3·75 ± 1.68 cm^2^; *P* =< 0.0001). For obvious reasons, the QLQ-CIPN20 score was not calculated in H_C_. On multivariate linear regression, increasing age had a significant (*P* = 0.01) correlation only with LDI_FLARE_ in H_C_ but not in any of the CIPN groups. Similarly, no significant effect of age was seen with other neural assessment methods, including VPT, SNCV, and SNCV in both H_C_ and CIPN groups.

There was no significant difference between the two CIPN groups, P_A_ and T_X,_ in respect to age, sex distribution, BMI, systolic, and diastolic blood pressure (Table 1997). Both groups comprised patients with similar duration of history following completion of chemotherapy regimens (*P* = 0.98). In both CIPN groups, there was no significant difference in neurophysiological parameters, including VPT (*P* = 0.67), SNAP (*P* = 0.29) and SNCV (*P* = 0.25). In contrast, SNF function assessed by the LDI_FLARE_ method was significantly reduced in the P_A_ group compared to the T_X_ group (2.72 ± 0.54 vs. 4.79 ± 1.99 cm^2^; *P* = 0.007). Furthermore, symptomatology and functional impairment measured using the QLQ-CIPN20 questionnaire was significantly worse as shown by higher scores in P_A_ group compared to the T_X_ group (48.08 ± 7·.40 vs. 29.25 ± 7.21 cm^2^; *P* =< 0.0001).

**Figure 3:**
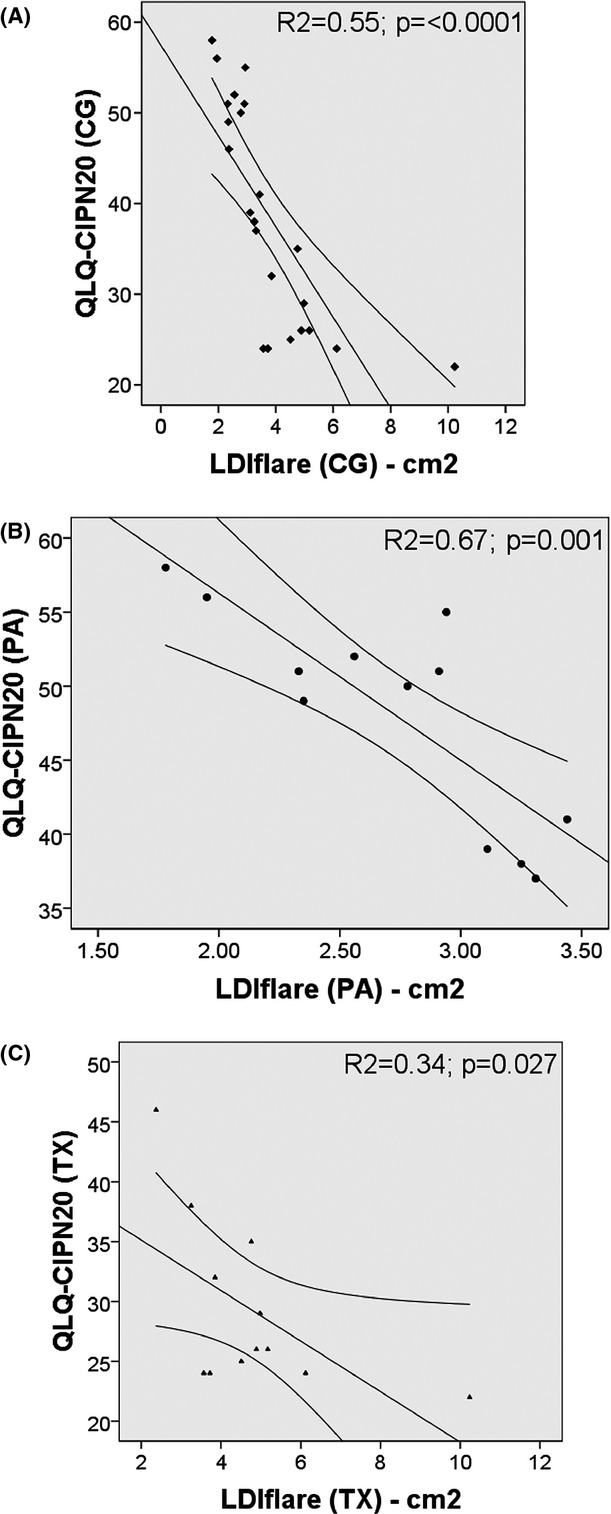
Shows the correlations and their significance when the QLQ-CIPN20 score and LDI_FLARE_ are compared in Combined (C_G_) (A), Platinum analogs (P_A_) (B), and Taxanes (T_X_) groups (c).

**Figure 4:**
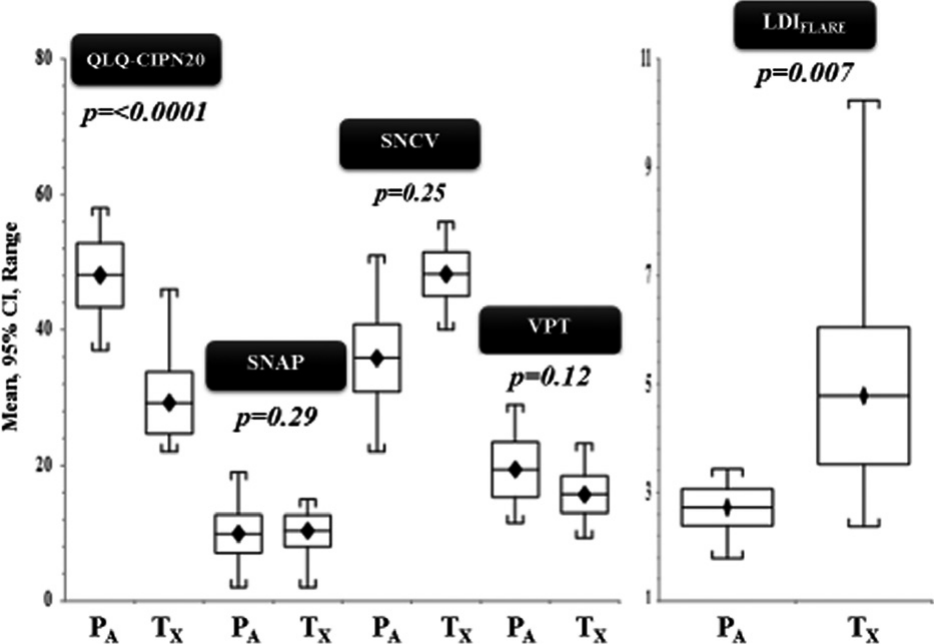
Shows significance differences in QLQ CIPN20 scores and LDI_FLARE_ between Platinum analogs (P_A_), and Taxanes (T_X_) and the absence of significance with other neurophysiological tests (SNAP, SNCV and VPT).

There was no relation between the QLQ-CIPN20 scores and large fibre modalities (VPT, SNAP, and SNCV) (Table 1997). The QLQ-CIPN20 clinical score showed a good correlation with small fibre function (LDI_FLARE_) in the overall C_G_ group (Fig.3A) as well as within the P_A_ (Fig.3B) and T_X_ (Fig.3C) groups individually. Indeed, after removing an extreme outlier with a flare size of 10.23 cm^2^ who had the lowest QLQ-CIPN20 score (22/80), the significance of the correlation with the LDI_FLARE_ was substantially greater (*P* = 0.001). In contrast, the box plots shown in Fig.4 depicting the distribution of large and small fibre parameters in individual P_A_ and T_A_ groups demonstrate that there is no difference in large fibre parameters between these groups, whereas small fibre function reflected inLDI_FLARE_ was significantly reduced in P_A_ when compared to T_A_.

## Discussion

The diagnosis and quantification of CIPN can be challenging for the reasons already stated. Physician-based scoring methods are limited by wide variations between the physicians' individual assessments as well as differences between these assessments and patients' self-reporting questionnaires (Windebank and Grisold 2008). For these reasons, none of the currently available grading scales have been universally accepted and indeed, this may account for the wide variety of questionnaires that have been developed for use in studies of different chemotherapeutic agents (Cavaletti et al. 2010). To support the physician/patient-based scoring scales, QST, EPM measurements (NCV, EMG) and nerve biopsy are often used. However, they do not consistently correlate with patients' symptoms and functional impairment (Forsyth et al. 1997). Moreover, these methods do not permit early detection of small fibre dysfunction which may be important in predicting progression to CIPN which in turn could influence therapeutic changes, including dose modification, treatment interruption or even discontinuation of chemotherapy (du Bois et al. 1999). Indeed, Hausheer et al. (2006) state “… none of these assessments would meet the criteria we have proposed for diagnosis, management, and reliability in this patient population, either in practice or as endpoint assessments of CIPN”. These conclusions are perhaps not surprising as methods like VPT and EPM measurements (NCV, EMG) assess large myelinated A*α*-*β* nerve fibres serving motor and sensory innervation but do not assess the function of small myelinated A-*δ* and unmyelinated C fibres, which serve pain and thermal sensations, the disturbance of which is the hallmark of early CIPN.

In contrast to the above methods, the LDI_FLARE_ selectively and quantitatively assesses SNF function by measuring the size of the C-fibre-mediated vasodilatory flare. We have shown the LDI_FLARE_ to be a reliable and sensitive technique of assessing SNF function in a variety of diabetic states (Krishnan and Rayman 2004; Green and Rayman 2007). In the present study, the LDI_FLARE_ has shown SNF to be significantly reduced in C_G_ as well as in the individual sub-groups P_A_ and T_X_ groups when compared to matched-H_C_. In contrast, and in keeping with many other studies, EPM indices (SNAP and SNCV) were not significantly different in the C_G_ group compared to H_C_, nor between P_A_ and T_X_ sub-groups. This suggests that the LDI_FLARE_ technique is more specific and superior to EPM methods in detecting CIPN and supports the importance of assessing SNF over tests of large fibre function.

It might be argued that nerve biopsy would be the most definitive investigation to diagnose early CIPN. However to date, there is limited nerve biopsy data in humans, the majority of studies relate to rodent experimental models. Hence, in general, except in atypical cases, nerve biopsy for CIPN is rarely indicated in clinical practice (Cavaletti et al. 2008). Skin biopsy with measurement of intra-epidermal nerve fibre density (IENFD) is a viable alternative; however it is infrequently used in the diagnosis of CIPN partly because it is invasive and expensive (Lauria 2005; Windebank and Grisold 2008). Furthermore, multiple biopsies which patients may not accept, would be required if progression is to be studied. In contrast, the LDI_FLARE_ technique has been shown to be as sensitive as IENFD in detecting early small fibre damage (Krishnan et al. 2009) and has the added advantage of being noninvasive and therefore more suitable for repeated studies. Moreover, it assesses nerve function which is more likely to be impaired before there is the degree of structural damage to be reflected by a reduced IENFD.

An important finding in this study is the correlation of the LDI_FLARE_ technique with patient symptom-scores not only for the C_G_ but also in each of the chemotherapeutic sub-groups. In contrast, neither of the other methods used in the study correlated with symptom-scores in C_G_ nor with those in the individual groups – P_A_ and T_X_. The lack of correlation with existing methods is in keeping with many previous studies (Hausheer et al. 2006). This not surprising, as in contrast to the LDI_FLARE_ technique, these methods mainly assess large fibre function, while symptoms like hyperpathia, hyperalgesia, and allodynia predominantly relate to SNF dysfunction.

Another important observation in this study was the ability of the LDI_FLARE_ technique to differentiate severity of CIPN between the two subgroups P_A_ and T_X_ (Fig.4). The P_A_ cohort had significantly higher QLQ-CIPN20 scores when compared to T_X_. This difference in severity is supported by significantly smaller LDI_FLARES_ in P_A_ as compared to T_X_ (2.72 ± 0.54 vs. 4.79 ± 1.99 cm^2^; *P* = 0.005). In contrast, all three large fibre modalities (VPT, SNAP, and SNCV) failed to show any significant differences in between the two different classes of chemotherapy. Based on this, we suggest that the LDI_FLARE_ technique is a more powerful tool to quantify and differentiate the severity of CIPN between these chemotherapy drugs.

This study is not without its limitations. Firstly, we recognize that the study has a relatively small number of patients in each group. However, we are confident that based on these highly significant findings, we would expect the same to be confirmed in larger studies. Secondly, we used only the EORTC QLQ-CIPN20 questionnaire rather than any of the other available questionnaires many of which are specific for platinum- or taxane-related CIPN. The reason for choosing the QLQ-CIPN20 questionnaire was to adopt a widely accepted and validated scoring system that could be applied to both treatment groups, that is, P_A_ and T_X_. Thirdly, since the LDI_FLARE_ method was tested on a cohort of subjects who already had established CIPN disease, it would be premature to comment that this method is superior to other measures of neural function or structure in assessing early CIPN disease.

In summary, our findings suggest that the LDI_FLARE_ technique is an effective method for confirming that patients' subjective symptoms are related to small fibre neuropathy and offers better investigative potential in the diagnosis and quantification of CIPN than existing methods. Furthermore, it correlates well with patients' symptoms and functional impairment, suggesting that it may be useful when combined with patient symptom-scores in modifying chemotherapy posology. Future prospective studies using the LDI_FLARE_ technique are required in treatment-naïve patients' to track changes of SNF function during treatment and to determine whether this technique is helpful in early identification of those at risk of developing CIPN.
